# Dihydromyricetin Inhibits Pseudorabies Virus Multiplication In Vitro by Regulating NF-κB Signaling Pathway and Apoptosis

**DOI:** 10.3390/vetsci10020111

**Published:** 2023-02-02

**Authors:** Xufan Zhao, Yaqin Chen, Wenrui Zhang, Hui Zhang, Yilong Hu, Fengyu Yang, Yingying Zhang, Xu Song

**Affiliations:** 1 Natural Medicine Research Center, College of Veterinary Medicine, Sichuan Agricultural University, Chengdu 611130, China; 2 College of Animal Science and Technology, Sichuan Agricultural University, Chengdu 611130, China

**Keywords:** pseudorabies virus, dihydromyricetin, antiviral activity

## Abstract

**Simple Summary:**

Pseudorabies virus (PRV) is a herpesvirus with zoonotic potential and has caused significant economic losses to the global pig industry. With the emergence of new mutants, the immune protection provided by vaccines has been greatly reduced, and it has become more difficulty to control PRV by vaccination. Recently, human cases of PRV-induced encephalitis have been reported, which suggest an urgent need for new control measures. In this study, we found that dihydromyricetin (DMY) exerted potent antiviral activity against PRV in vitro. DMY could ameliorate the PRV-induced abnormal activation of the NF-κB signaling pathway and excessive cellular inflammatory response. DMY could also induce the apoptosis of PRV-infected cells, thereby limiting the production of progeny virus. Based on the findings, DMY could be a candidate drug for the treatment of PRV infections.

**Abstract:**

Pseudorabies virus (PRV) infections have caused huge economic losses to the breeding industry worldwide, especially pig husbandry. PRV could threaten human health as an easily ignored zoonotic pathogen. The emergence of new mutants significantly reduced the protective effect of vaccination, indicating an urgent need to develop specific therapeutic drugs for PRV infection. In this study, we found that dihydromyricetin (DMY) could dose-dependently restrain PRV infection in vitro with an IC50 of 161.34 μM; the inhibition rate of DMY at a concentration of 500 μM was 92.16 %. Moreover, the mode of action showed that DMY directly inactivated PRV virion and inhibited viral adsorption and cellular replication. DMY treatment could improve PRV-induced abnormal changes of the NF-κB signaling pathway and excessive inflammatory response through regulation of the contents of IκBα and p-P65/P65 and the transcriptional levels of cytokines (TNF-α, IL-1β and IL-6). Furthermore, DMY promoted the apoptosis of PRV-infected cells through the regulation of the expressions of Bax and Bcl-xl and the transcriptional levels of Caspase-3, Bax, Bcl-2 and Bcl-xl, thereby limiting the production of progeny virus. These findings indicated that DMY could be a candidate drug for the treatment of PRV infection.

## 1. Introduction

Herpesviruses, as a large class of dsDNA viruses that can be widely transmitted in a variety of organisms, can produce severe viral diseases in both humans and animals [1]. Pseudorabies virus (PRV), commonly recognized as the suid herpesvirus 1 [2], is a porcine alphaherpervirus [3]. PRV could also be a zoonotic pathogen that is easily overlooked [4]. Most reports of PRV infections appear in animals, but the number of human cases has gradually increased, and China has reported 25 cases of human infection with PRV in the last five years [5], which warrants closer investigation, as it could be dangerous to human health.

PRV has a widely transmitted reservoir that causes disease in a variety of domestic animals as well as wild animals [6,7], and the primary reservoir of the virus is pig [8]. The infection of susceptible animals is usually fatal, and the symptoms after infection are more obvious, primarily including fever, itching (except pigs) and central nervous system symptoms [9]. As a highly contagious and lethal pathogen, it has contributed to tremendous economic losses to the global breeding industry, especially the pig industry [10]. PRV has neurotropic properties similar to its subfamily members, leading to latent infection in the central and peripheral nervous systems [11], and the latently infected host can carry the virus for life, and which can be activated under favorable conditions [12]. With neuroinfective characteristics, PRV is also serving as an ideal mode virus to test the antivirals against viral encephalitis [13]. With the emergence of virus variants, the cases of human infected with PRV and even transmission between humans have also increased [14]. Human infection mainly causes central nervous system symptoms, viral encephalitis, optic nerve symptoms and necrotizing retinitis, which is life-threatening [15,16]. Currently, there is no specific treatment drug for PRV, and prevention is mainly through vaccination. However, PRV has a high level of genomic variation, leading to the emergence of new mutants, which greatly reduces the preventive efficacy of classical vaccines [17,18]. At present, the most classic drugs for the treatment of herpesvirus infection are nucleoside drugs, including acyclovir, ganciclovir and adefovir dipivoxil [19]. However, these medications can only affect the virus during its replication and lysis phase and have no effect on the latent virus. Additionally, the prolonged use of these medications will lead to the emergence of drug-resistant strains, which makes them unsuitable for long-term clinical use [20].

**Figure 1:**
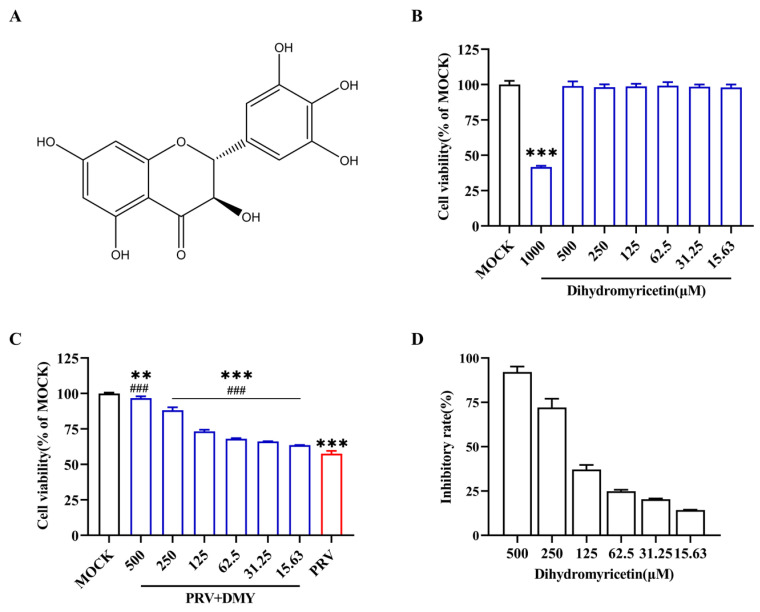
Antiviral activity of dihydromyricetin against PRV. (**A**) The constructive formula of dihydromyricetin (DMY). (**B**) Toxicity of DMY on PK-15 cells. Two-fold dilutions of DMY (1000–15.63 μM) were incubated with PK-15 cells, and a CCK-8 kit was utilized to assess cell viability after incubation for 48 h. (**C**) Cell viability after infection with PRV. The PK-15 monolayer was subjected to 100 TCID_50_ PRV in conjunction with DMY for 1 h before being switched to a maintenance medium containing DMY. Once more, the CCK-8 kit was used to conduct the test after 48 h. (**D**) DMY’s resistance to PRV inhibition rate. Inhibition rate = (OD 450 nm value of DMY-treated infected cells—OD 450 nm value of untreated infected cells) ÷ (OD 450 nm value of untreated uninfected cells—OD 450 nm value of untreated infected cells). Mock, normal cell groups; PRV, virus-inoculated group; PRV + DMY, virus-inoculated and treated group. ### indicates *p* < 0.001 compared to PRV group; *** indicates *p* < 0.001 compared to MOCK group.

Flavonoids, polyphenolic compounds that are widely found in nature, are widely investigated due to their diverse biological functions [21], such as anti-inflammatory, antioxidant, antibacterial, antiviral, anticancer and neuroprotective activities [22]. Flavonoids can affect specific steps in the life cycle of different viruses [23] and inhibit herpesvirus infection [24]. Therefore, flavonoids are a considerable source of potential anti-herpesvirus drugs [25,26]. Dihydromyricetin (DMY, Figure 1A) belongs to the class of flavonols, and was first extracted from *Ampelopsis meliaefolia* [27] and widely exists in a variety of plants, such as *Hovenia dulcis*, bayberry and ginkgo [28]. Studies have found that DMY has various biological activities, such as the induction of melanoma cell apoptosis by regulation of the ROS and NF-κB pathway [29] and protection of HUVECs from damage by the Nrf2/HO-1 pathway [30]. DMY can effectively inhibit HSV-1 replication by reducing expressions of HSV-1-related genes and reducing the production of TNF-α through the TLR9 pathway [31]. In this study, we tested the anti-PRV activity of DMY in vitro and evaluated the regulating effects on abnormal inflammation and apoptosis caused by PRV, which provided ideas for the development of new anti-herpesvirus drugs.

## 2. Materials and Methods

### 2.1. Compounds

Dihydromyricetin (98% HPLC purity; Meilunbio, Dalian, China) was stored at 4 °C in the dark. For antiviral tests, the DMY was dissolved in DMSO (Solarbio, Beijing, China), followed by dilution of at least 100-fold in DMEM (Gibco, CA, USA).

### 2.2. Cells and Virus

The PK-15 cells, purchased from the China Center for Type Culture Collection (Wuhan, China), were grown in DMEM containing 10% (*v*/*v*) fetal bovine serum (PAN, Germany), penicillin (100 U/mL), and streptomycin (100 μg/mL). For cell maintenance, the content of fetal bovine serum was reduced to 2%. The Ra strain of PRV was purchased from the China Veterinary Culture Collection (Beijing, China). The virus was proliferated in PK-15 cells. The 50% tissue culture infectious dose (TCID_50_) was determined to be 10^−7.1^ mL^−1^ [32].

### 2.3. Cytotoxicity and Inhibitory Activity Assays

The cytotoxicity of DMY and the inhibitory activity against PRV were reflected by cell viability, which was measured by a Cell Counting Kit-8 (CCK-8; Meilunbio, Dalian, China). Cytotoxicity was determined by the co-incubation of monolayers with two-fold dilutions of DMY ranging from 1000 to 15.63 μM in 96-well plates. After incubation for 48 h, CCK-8 solution (10 μL) was added, followed by incubation for 30 min. The absorbance of the plates at 450 nm was detected using a microplate reader (BioRad, Hercules, CA, USA). For determining the antiviral effect of DMY, the virus suspension (100 TCID_50_) was added into PK-15 monolayer in the presence or absence of DMY. The mixed culture medium was then removed after incubation for 1 h. After washing, DMY was added again, followed by re-incubation for 48 h. The absorbance of the plate at 450 nm was determined by the CCK-8 kit. Finally, the median toxic concentration (CC_50_) of DMY on PK-15 cells and the median inhibitory concentration (IC_50_) against PRV were calculated by the Reed-Muench method.

### 2.4. Inhibitory Action Assay

Cell pretreatment assay: DMY was added into the cell monolayer, followed by incubation at 37 °C for 1 h. After removal of the medium, the virus suspension (100 TCID_50_) was added at 37 °C for 1 h. After washing, cells were incubated for 48 h, and then the virus titer was detected by the fluorescent quantitative PCR (FQ-PCR) method based on the gB gene [33]. The total viral DNA of infected cells was extracted with a virus genome extraction kit (Biomed, Beijing, China). The primers were 5′-ACAAGTTCAAGGCCCACATCTAC-3′ (Forward) and 5′-GTCYGTGAAGCGGTTCGTGAT-3′ (Reverse), and the probe was 5′-ACGTCATCGTCACGACC-3′. The FQ-PCR reaction conditions were 95 °C for 120 s, 95 °C for 5 s, and 56.5 °C for 30 s (40 cycles), which was performed on a Bio-Rad CFX96 Connect^TM^ Real-Time PCR Detection System (CA, USA).

Inactivation assay: DMY were co-incubated with 10000 TCID_50_ PRV. After incubation at 37 °C for 1 h, the mixture was diluted by 100-fold and added into cells for 1 h incubation. After washing, maintenance medium was added for 48 h. Finally, the total DNA of each group was extracted and FQ-PCR was performed.

Adsorption assay: Virus suspension (100 TCID_50_) was added into cells in the presence of DMY at 4 °C for 1 h. Then, the virus-containing mixture was discarded and maintenance medium was added for 48 h at 37 °C. The total DNA was extracted and FQ-PCR was conducted.

Penetration assay: The cell monolayer was pre-cooled at 4 °C for 30 min, followed by infection with 100 TCID_50_ PRV at 4 °C for 1 h. After washing with pre-cooled PBS, maintenance medium containing DMY was added and the cells were incubated at 37 °C for 1 h. The cells were then washed with a citric acid-sodium citrate buffer (pH = 3.0) for the inactivation of unpenetrated virus. The maintenance medium was added at 37 °C for 48 h. The total DNA was extracted and FQ-PCR was performed.

Replication assay: The cells were infected with 100 TCID_50_ PRV at 37 °C for 1 h, and then the medium was removed. DMY-containing maintenance medium was added, and after culturing at 37 °C for 48 h, total DNA was extracted and FQ-PCR was conducted.

Growth curve assay: Virus suspension (0.1 MOI) was added into the PK-15 monolayer at 37 °C for 1 h. After washing, DMY-containing maintenance medium was added, and the cells were collected at 2 h, 4 h, 6 h, 8 h, 12 h, 18 h, and 24 h, respectively. The total DNA was extracted and FQ-PCR was conducted.

### 2.5. Transcriptional Levels of Target Genes Assay

The cells and virus were treated as described in the growth curve assay. The total RNA was extracted by TRzol reagent (Biomed, Beijing, China), followed by reverse transcription (M-MLV 4 First-Strand cDNA Synthesis Kit; Biomed, Beijing, China). The RT-PCR was conducted with primers of the tested genes (Table 1) by a Hieff UNICON^®^ qPCR SYBR Green Master Mix (Yeasen, Shanghai, China). The PCR cycles were 95 °C for 3 min; 95 °C for 10 s, 59.8°C for 30 s, and 55°C for 5 s (40 cycles). Finally, a melting curve analysis was performed. The expression level of β-actin was used to normalize the difference in the expression of each target gene, and 2^^-△△Ct^ was used to calculate the relative expression level of each gene.

### 2.6. Western Blotting

PK-15 cells were incubated with virus suspension (MOI = 1) for 1 h, and, after washing, maintenance medium with or without DMY was added. Total intracellular protein was extracted at 4 h, 8 h, 12 h, 18 h, and 24 h, respectively. The proteins were isolated through SDS-PAGE, and then transferred onto a PVDF membrane (Millipore, USA). The membrane was blocked in 5% (w/v) skim milk for 90 min at RT, and then different antibodies were added, including P65 (CST, USA, 1:1000), p-P65 (Bioss, Beijing, 1:2000), IκBα (CST, USA, 1:2000), Bax (Proteintech, Wuhan, China, 1:1000), Bcl-xl (Proteintech, Wuhan, China, 1:1000), and β-actin (Bioss, Beijing, China, 1:2000). The membranes were incubated overnight at 4°C. After washing, horseradish peroxidase-conjugated secondary antibody was added for 1 h at RT. Finally, proteins were visualized through enzymatic chemiluminescence reagents (ECL, Biosharp, Anhui, China). The expression of total protein was normalized by β-actin.

### 2.7. Statistical Analysis

The data are presented as the mean ± standard deviation. Statistical significance was determined by one-way analysis of variance using SPSS 21.0 Statistical Software (IBM, NY, USA), and *p* < 0.05 was considered statistically significant.

## 3. Results

### 3.1. Results

#### 3.1.1. Cytotoxicity and Antiviral Activity of Dihydromyricetin

In the present study, DMY was diluted with DMEM in order to reduce the final concentration of DMSO to no more than 0.5%, which had no effects on the cells or the virus. The cytotoxicity of DMY in PK-15 cells was detected by CCK-8, and the cell viability was not significantly changed when the concentration of DMY ranged from 500 to 15.63 μM (Figure 1B). The CC_50_ was calculated as 914.32 ± 1.19 μM (Table 2). DMY ranging from 500 to 15.63 μM exhibited a dose-dependent antiviral activity against PRV (Figure 1C). The inhibition rate was 92.16 % when the concentration of DMY was 500 μM (Figure 1D). The IC_50_ of DMY was calculated to be 161.34 ± 0.86 μM, and its selectivity index (SI) was 5.68 (Table 2).

#### 3.1.2. Effect of Dihydromyricetin on Specific Steps of Viral Life Cycle

**Figure 2:**
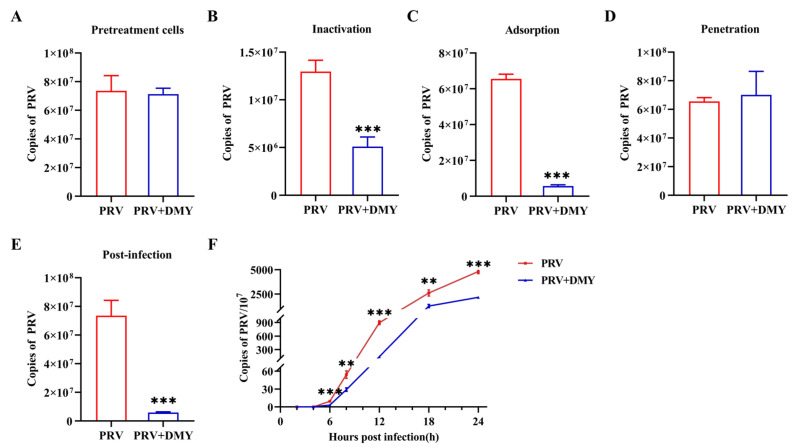
DMY’s mechanism of action against PRV. (**A**) The pretreatment effect of DMY against PRV. (**B**) The viral inactivation effect of DMY. (**C**) The inhibitory effect of DMY on the viral adsorption process. (**D**) Inhibition of PRV penetration by DMY. (**E**) Inhibition of DMY against PRV intracellular replication. (**F**) PRV growth curve. Viral DNA copy number at each stage was detected by FQ-PCR. PRV, virus-inoculated group; PRV + DMY, virus-inoculated and treated group, the concentration of DMY was 500 μM. Compared with the PRV group, symbols *, ** and *** denote *p* < 0.05, *p* < 0.01 and *p* < 0.001, respectively.

In order to further determine the antiviral mechanism of DMY in PK-15 cells, five independent experiments were set up to detect the viral replication characteristics. In the cytoprotective assay (pretreatment), DMY was added to the cells first and then infected with PRV, but no anti-PRV effect was detected (Figure 2A). In the inactivation assay, DMY was co-incubated with PRV and then added to the cells. It was found that DMY treatment could significantly reduce the virus copy number (Figure 2B). In the adsorption assay, DMY and PRV were simultaneously added to the cells at 4°C, and DMY significantly inhibited the adsorption process of PRV (Figure 2C). After virus attaches to the cell surface, the penetration process occurs, but DMY does not show inhibitory activity against PRV during this process (Figure 2D). After the virus penetrated into the cell, it began to replicate itself using the raw materials and energy system provided by the host cell. We found that DMY could significantly inhibit the replication of PRV (Figure 2E). In addition, we detected the virus growth curve during the intracellular replication process within 24h after the addition of DMY. DMY treatment did not inhibit the early stage of infection within 4 h post infection (hpi), but could significantly inhibit viral proliferation from 6 to 24 hpi (Figure 2F).

#### 3.1.3. Effect of Dihydromyricetin on PRV-Activated NF-κB Signaling Pathway

**Figure 3:**
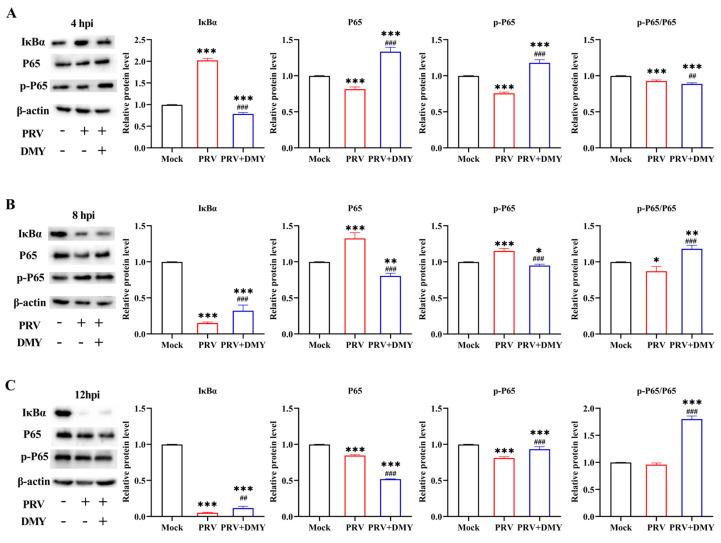
The effects of dihydromyricetin on the NF-κB signaling pathway during 4 hpi (**A**), 8 hpi (**B**), and 12 hpi (**C**). PK-15 cells treated or untreated with DMY (500 μM) were infected with PRV (MOI = 1), and the expressions of pathway-related proteins, including Iκ-Bα, P65 and p-P65, were examined by western blotting. Mock, normal cell groups; PRV, virus-inoculated group; PRV + DMY, virus-inoculated and treated group. Compared with the mock group, symbols *, ** and *** denote *p* < 0.05, *p* < 0.01 and *p* < 0.001, respectively. Compared with the PRV group, symbols ## and ### denote *p* < 0.01 and *p* < 0.001, respectively. The uncropped western blot in Supplementary Materials Figure S1.

For facilitating viral proliferation after infection, related signaling pathways are usually changed by the virus, such as the classical NF-κB signaling pathway. At 4 hpi, the expression of IκBα was significantly increased after PRV-infection, and PRV also significantly decreased the levels of P65, p-P65 and p-P65/P65 in comparison with blank control. DMY-treatment significantly increased the expressions of P65 and p-P65, and the levels of IκBα and p-P65/P65 were significantly decreased (Figure 3A). At 8 hpi, PRV-infection significantly increased the expressions of P65 and p-P65, and the levels of IκBα and p-P65/P65 was significantly decreased in comparison with the blank control group. DMY-treatment significantly increased the levels of IκBα and p-P65/P65; the levels of P65 and p-P65 were significantly decreased (Figure 3B). At 12 hpi, the levels of IκBα, P65 and p-P65 were significantly decreased in the PRV-infected group. The levels of IκBα, p-P65 and p-P65/P65 were significantly enhanced in the DMY-treated group, while the p65 level was significantly decreased (Figure 3C).

#### 3.1.4. Effect of Dihydromyricetin on PRV-Changed Cell Apoptotic Process

**Figure 4:**
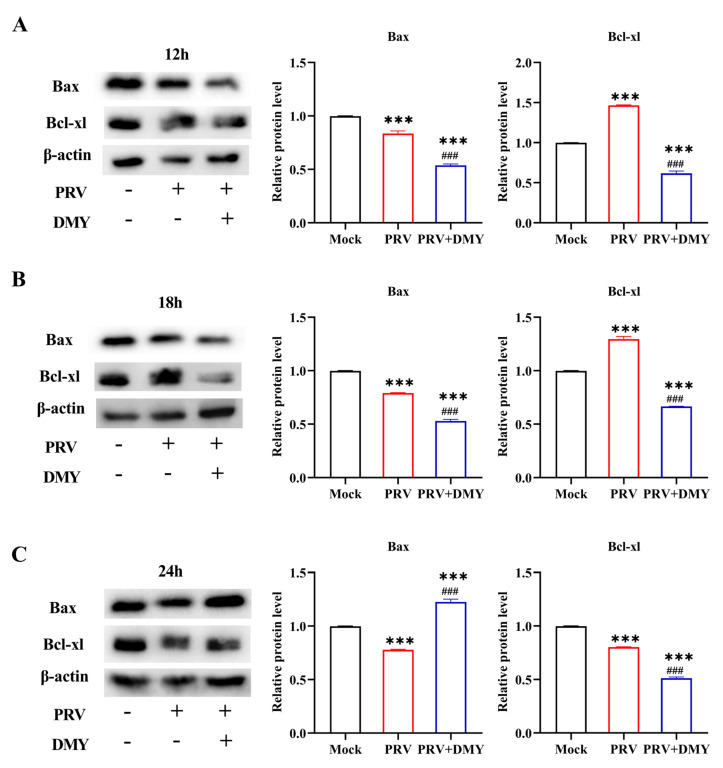
The impact of dihydromyricetin on the expression of the apoptosis-inducing protein at 12 (**A**), 18 (**B**), and 24 (**C**) hpi after infection. In the presence or absence of 500 μM DMY, PRV (MOI = 1) was added to PK-15 cells. Western blotting was used to identify the expressions of Bax and Bcl-xl. Mock, normal cell groups; PRV, virus-inoculated group; PRV + DMY, virus-inoculated and treated group. Compared with mock group, Symbol *** denotes *p* < 0.001. Compared with PRV group, symbol ### denotes *p* < 0.001. The uncropped western blot in Supplementary Materials Figure S2.

PRV infection can cause a variety of host responses, including interference with the normal apoptosis process of host cells, so we also tested the expressions of key apoptosis proteins, Bax and Bcl-xl (Figure 4). In comparison with the normal group, the PRV-infected group showed a significantly higher Bcl-xl level and lower Bax level at 12 hpi. After treatment with DMY, the levels of Bax and Bcl-xl in PRV-infected cells were significantly down-regulated (Figure 4A). At 18 hpi, PRV infection significantly up-regulated the Bcl-xl level and down-regulated the Bax level; the levels of Bax and Bcl-xl in the DMY-treated group were significantly down-regulated (Figure 4B). At 24 hpi, the PRV-infected group exhibited significantly lower levels of Bax and Bcl-xl; while DMY treatment significantly up-regulated the Bax level and significantly decreased the Bcl-xl level (Figure 4C).

#### 3.1.5. Effect of Dihydromyricetin on Gene Expressions of Cytokines and Apoptotic Factors

**Figure 5:**
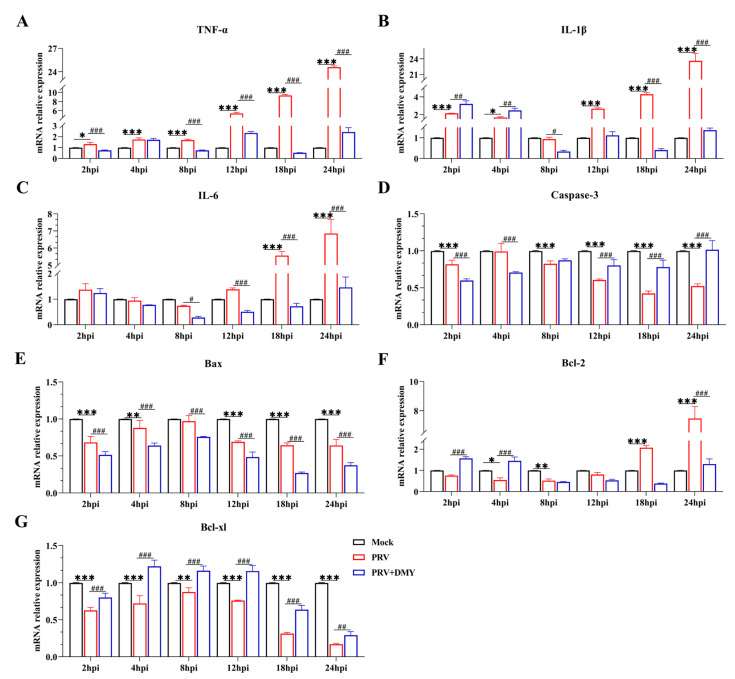
Effects of dihydromyricetin on gene expressions at 2, 4, 8, 12, and 24 hpi. The NF-κB signaling pathway’s genes involved are TNF-α, IL-1β, and IL-6. Caspase-3, Bax, Bcl-2, and Bcl-xl are apoptotic factors. Mock, uninfected-untreated group; PRV, infected-untreated group (MOI = 1); PRV + DMY, infected-treated group; the concentration of DMY was 500 μM. Symbol *** denotes *p* < 0.001 when compared with mock group. Symbols *, **, and *** denote *p* < 0.05, *p* < 0.01 and *p* < 0.001, in several, as compared to the mock group. When compared to the PRV group, Symbols #, ##, and ### indicate *p* < 0.05, *p* < 0.01 and *p* < 0.001, respectively.

DMY could regulate the PRV-induced changes of the NF-κB pathway, and thus the expressions of downstream target genes (TNF-α, IL-1β and IL-6) were detected to further determine the activation of the signaling pathway (Figure 5). We found that PRV-infection induced significantly higher transcriptional levels of TNF-α, IL-1β and IL-6, and that DMY-treatment exhibited significantly lower mRNA levels of these cytokines. It was found that PRV infection significantly inhibited the levels of Caspase-3, Bax and Bcl-xl within 24 hpi, but the Bcl-2 level was significantly enhanced at 18 and 24 hpi. After treatment with DMY, the higher expressions of Caspase-3 and Bcl-xl were detected, but lower levels of Bax and Bcl-2 were found.

### 3.2. Figures and Tables

**Table 1:** Primer sequences for real-time PCR.

Primer	Forward (5′→3′)	Reverse (5′→3′)
β-actin	GGACTTCGAGCAGGAGATGG	AGGAAGGAGGGCTGGAAGAG
TNF-α	GAGATCAACCTGCCCGACT	TCACAGGGCAATGATCCCAA
IL-1α	AGAATCTCAGAAACCCGACTGTTT	TTCAGCAACACGGGTTCGT
IL-1β	GCCCTGTACCCCAACTGGTA	CCAGGAAGACGGGCTTTTG
IL-6	ATTAAGTACATCCTCGGCAAA	GTTTTCTGCCAGTACCTCC
Bax	GTTTCATCCAGGATCGAGCA	TGCAGCTCCATGTTACTGTCC
Caspase-3	AAGACCATAGCAAAAGGAGCA	GTTCACAGCAGTCCCCTC
Bcl-2	CTGCACCTGACTCCCTTCACC	TCCCGGTTGACGCTCTCCACA
Bcl-xl	GCCACTTACCTGAATGACCA	ATTGTTTCCGTAGAGTTCCAC

**Table 2:** Anti-PRV activity of dihydromyricetin.

Compound	CC_50_ (μM)	IC_50_ (μM)	SI
Dihydromyricetin	914.32 ± 1.19	161.34 ± 0.86	5.68

## 4. Discussion

PRV infection caused significant damages to animal health, and emerging virus variants can escape the immune protection induced by the classic vaccine, Bartha-K61 [34]. Moreover, the possibility of PRV becoming a threat to human health is gradually increasing due to the constant mutation of this virus [35]. Therefore, development of anti-PRV drugs is of great importance for the prevention and control of PRV infection [36]. It has been shown that myricetin, luteolin and kaempferol exhibited an inhibitory effect on herpesvirus infection. Myricetin blocks HSV infection by directly interacting with gD while down-regulating the EGFR/PI3K/Akt signaling pathway [37,38]. To prevent EBV reactivation, luteolin reduces the expressions of the promoters of Zta and Rta [39]. Kaempferol can inhibit PRV proliferation by regulation of the NF-κB and MAPK pathways [40,41]. Although several compounds with therapeutic effects against PRV have been identified, the underlying mechanisms are unknown, and therefore new specific drugs and strategies for the treatment of PRV infection are needed [42]. In this study, the selection index was used to measure the anti-PRV potency of DMY [33]. DMY had a selection index of 5.68 and dose-dependently inhibited PRV replication, indicating that DMY could be a potent inhibitor of PRV. As a typical herpesvirus [43], this study also indicated the potential of DMY to control the infections of other herpesviruses.

To further study the antiviral mechanism, the assays based on different stages of viral infection were tested. The results showed that DMY could significantly reduce virus titer during the stages of inactivation, adsorption and intracellular replication, and DMY inhibited virus proliferation in the medium and late stages of infection according to the growth curve. The proliferation of PRV in cells relies mainly on various viral proteins [44,45]. First, the membrane glycoprotein gC binds to the cell membrane heparan sulfate proteoglycan, and then the gD protein acts to bind to the cell membrane receptor to make the virus particles firmly adsorb on the cell membrane [46]. Under the combined action of gB, gH and gL, membrane fusion is promoted, viral proteins are released into the cytoplasm, and then the nucleocapsid enters the nucleus through microtubules [47]. Gene transcription and expression are initiated under the activation of the only immediate early gene (IE180) of the virus, thereby entering the replication cycle [48,49], and after two envelope coats, self-assembly is completed and transferred to the cell surface to be released by exocytosis [50]. Therefore, it is speculated that DMY can inhibit the protein function of gC or gD, leading to the prevention of the adsorption process between PRV and the cell membrane, thereby reducing the number of infected virus particles.

As an important part of the body’s innate immunity, the NF-κB signaling pathway is involved in a variety of biological processes [51,52]. In the process of virus infection, for the needs of self-proliferation, virions affect the NF-κB pathway through various mechanisms, thereby evading the host’s immune response [53]. The PRV-induced activation of the NF-κB pathway has some similarities with the activation of typical signaling pathways, but it also has its own characteristics [54]. The NF-κB signaling pathway mediated by TNF-α can be inhibited by the ICP0 protein of PRV [55], and the activation of this pathway can be substantially independent of the classical IκB kinase IKK while reducing the production of genes involved in negative feedback loops [56]. The expressions of essential proteins IκBα, P65 and p-P65 were measured, and we found that PRV boosted the production of the upstream protein IκBα at 4 hpi and then decreased at 8 and 12 hpi, implying that PRV blocked IκBα phosphorylation at the early stage of infection and then promoted it later on. The level of p-P65/P65 was always decreased, indicating that PRV inhibits the NF-κB signaling pathway independently of IκBα, which is consistent with a previous report that PRV can trigger the abnormal activation of the NF-κB pathway [57]. The p-P65/P65 level was declined at 4 hpi and increased at 8 and 12 hpi after DMY treatment, indicating that DMY could reverse the inhibitory state of the NF-κB pathway caused by PRV infection.

The NF-κB pathway, a representative pro-inflammatory pathway, is involved in inflammatory response mainly through expressions of pro-inflammatory genes, including cytokines, chemokines and adhesion molecules [58]. The infection of mice by PRV resulted in increased levels of key proinflammatory cytokines in the pathway [59]. In this study, the expressions of inflammatory factors (TNF-α, IL-1α and IL-6) showed a gradually increasing trend. The results are consistent with the phenomenon of a “cytokine storm” in mice infected with PRV, which is an excessive inflammatory response caused by the unbalanced secretion of pro-inflammatory cytokines and the imbalance of pro- and anti-inflammatory responses [60]. It has been reported that DMY showed an anti-inflammatory effect and could downregulate the level of inflammatory genes through the NF-κB pathway [61]. It was revealed that DMY significantly inhibited the gene expression levels of TNF-α, IL-1α and IL-6, which supported the view that DMY can enhance cellular immunity without causing excessive inflammatory responses in PRV-infected cells.

Viral infection can activate extracellular pathways mediated by TNF-α [62] and further activate Caspase-3, which causes cell apoptosis [63]. However, many viruses can inhibit the premature death of infected cells through anti-apoptotic mechanisms, thereby ensuring the process of virus proliferation and increasing the production of progeny virus [64]. PRV also shares this mechanism of action in apoptosis. The viral US3 protein kinase can inhibit the apoptosis of the infected cell by regulation of the PI3-K/Akt and NF-κB pathways without affecting the production of progeny viruses [65,66]. In addition, viral glycoprotein gE mediates ERK 1/2 activation in T lymphocytes after infection, thereby triggering an anti-apoptotic response [67]. Previous studies have found that DMY extracted from Ampelopsis grossedentata exerts in vitro anti-PRV effects through anti-pyroptosis effects, but the apoptosis response was not detected [68]. Therefore, the effects of DMY on host cell apoptosis after PRV infection was studied. The pro-apoptotic effector Bax, a critical effector of the mitochondrial apoptotic pathway, plays a core role in the apoptotic process by regulating the permeability of the outer mitochondrial membrane through the formation of apoptotic pores embedded in the membrane [69]. Members of the lymphoma apoptosis regulatory family are considered to be regulators of cell death, including Bcl-2, Bcl-xl, and MCL-1, acting as chemical inhibitors of the pro-apoptotic protein Bax, which inhibits the release of Bax and cytochrome c and exerts an anti-apoptotic effect [70,71,72]. In the present study, PRV infection increased the levels of anti-apoptotic factors Bcl-2 and Bcl-xl, and induced lower levels of pro-apoptotic factors Caspase-3 and Bax; DMY treatment facilitated the apoptosis of PRV-infected cells. These results demonstrated that DMY could suppress viral proliferation by promoting the apoptosis of infected cells, leading to the limitation of the production of progeny viruses [73].

## 5. Conclusions

Dihydromyricetin possesses potent anti-PRV activity. It can inhibit the proliferation of PRV by regulating the NF-κB signaling pathway and the expressions of apoptotic factors.

## Data Availability

Raw data are available upon request.

## Supplementary Materials

The following are available online at https://www.mdpi.com/article/10.3390/vetsci10020111/s1, Figure S1: Uncropped western blot bands for Figure 3; Figure S2: Uncropped western blot bands for Figure 4; Table S1: Intensity ratio of the Western blot bands in Figure 3; Table S2: Intensity ratio of the Western blot bands in Figure 4.
