# Supplementary material for: Dihydromyricetin Inhibits Pseudorabies Virus Multiplication In Vitro by Regulating NF-κB Signaling Pathway and Apoptosis
Source: Vet Sci. 2023 Feb 2;10(2):111. doi: 10.3390/vetsci10020111 (PMC9961748; doi:10.3390/vetsci10020111)
Supplement: Supplementary file 1 [file vetsci-10-00111-s001.zip › vetsci-2061688-supplementary.pdf]

Figure S1.a: Uncropped western blot bands for Figure 3

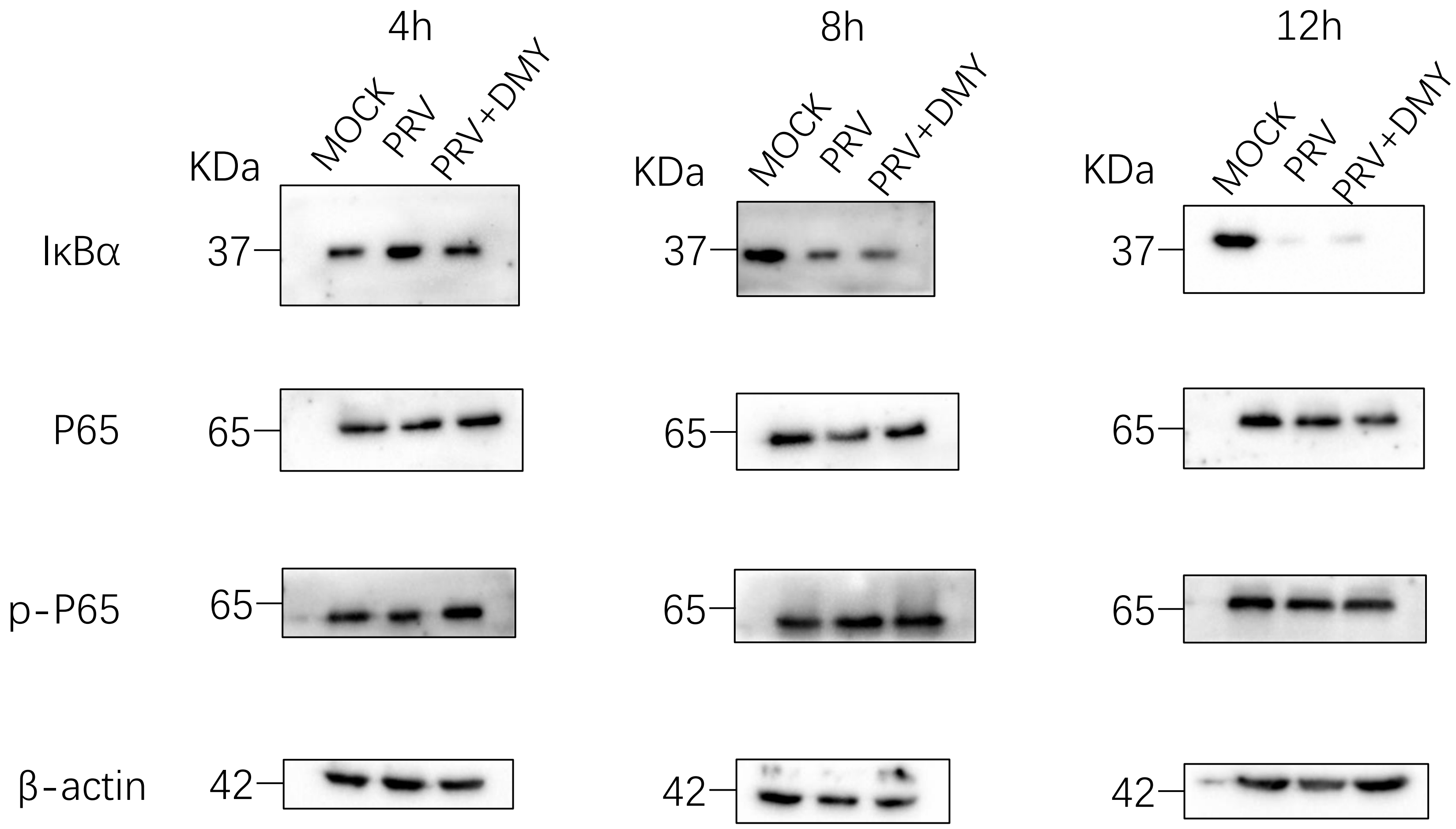

Figure S1.b

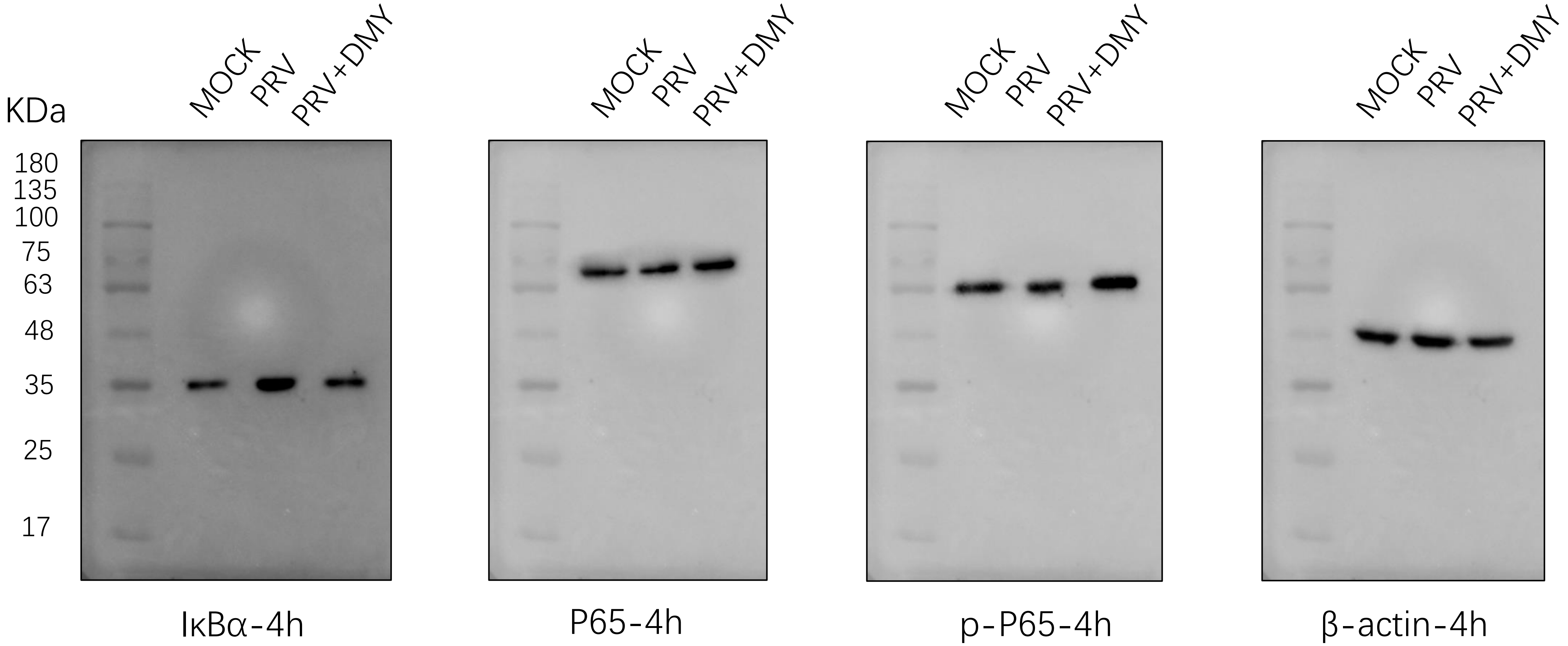
KDa
180
135
100
75
63
48
35
25
17
MOCK
PRV
PRV+DMY
IκBα-4h
P65-4h
p-P65-4h
β-actin-4h

Figure S1.c

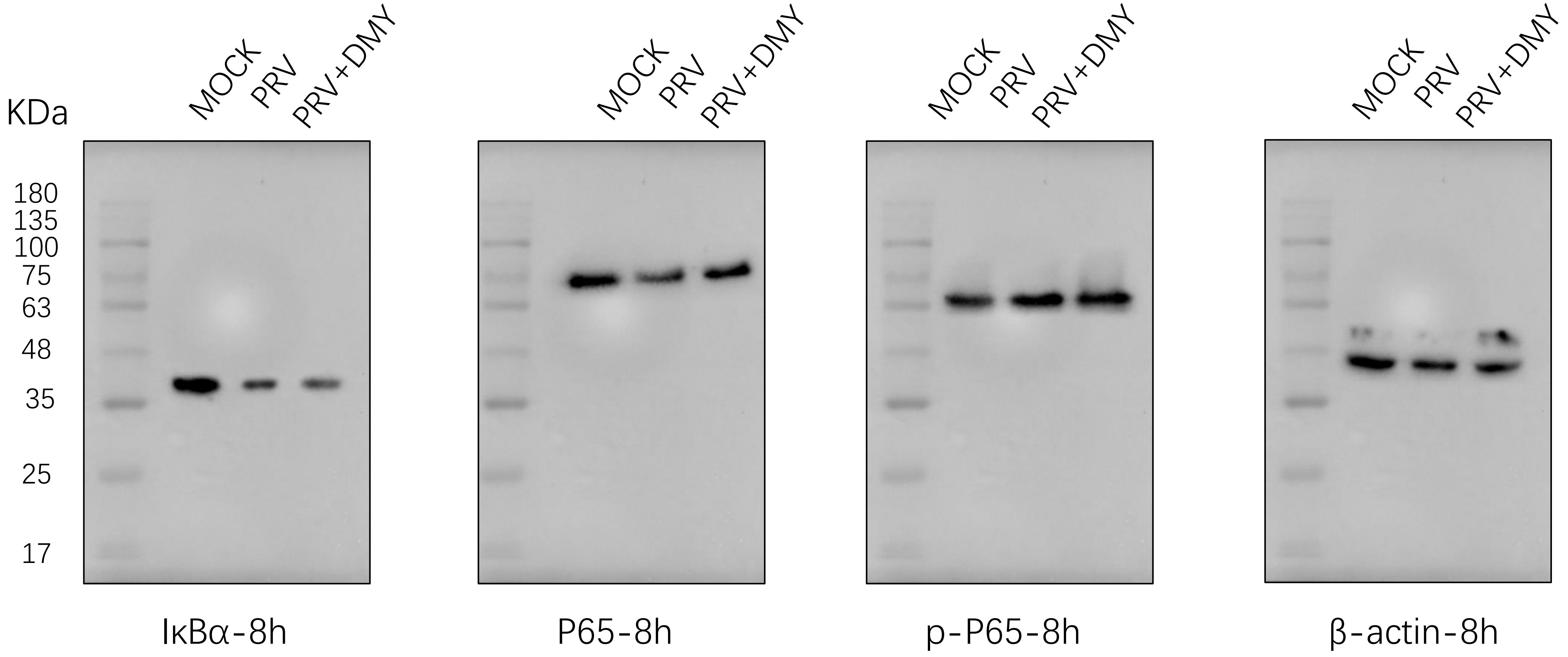
KDa
MOCK
PRV
PRV+DMY
180
135
100
75
63
48
35
25
17
IκBα-8h
P65-8h
p-P65-8h
β-actin-8h

Figure S1.d

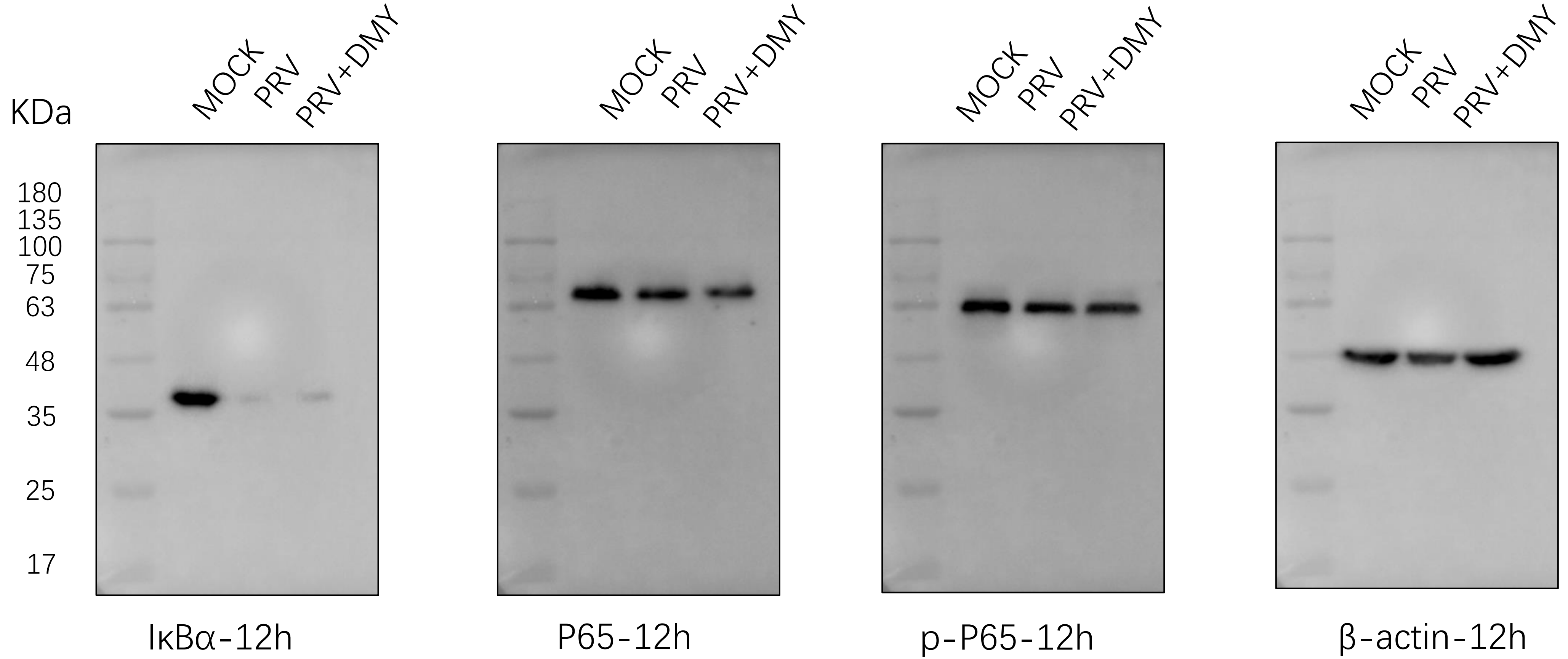
KDa
MOCK
PRV
PRV+DMY
180
135
100
75
63
48
35
25
17
IκBα-12h
P65-12h
p-P65-12h
β-actin-12h

Table S1: Intensity ratio of the Western blot bands in Figure 3

4h	Mock	PRV	PRV+DMY
IκBα	1	2.02±0.04	0.78±0.03
P65	1	0.81±0.02	1.33±0.05
p-P65	1	0.76±0.01	1.18±0.03
β-actin	1	1.01±0.09	0.97±0.05

8h	Mock	PRV	PRV+DMY
IκBα	1	0.15±0.04	0.32±0.06
P65	1	1.32±0.06	0.80±0.03
p-P65	1	1.15±0.03	0.95±0.02
β-actin	1	1.05±0.06	1.09±0.09

12h	Mock	PRV	PRV+DMY
IκBα	1	0.05±0.01	0.12±0.02
P65	1	0.85±0.01	0.52±0.01
p-P65	1	0.81±0.02	0.93±0.03
β-actin	1	1.04±0.05	1.05±0.07

Figure S2.a: Uncropped western blot bands for Figure 4

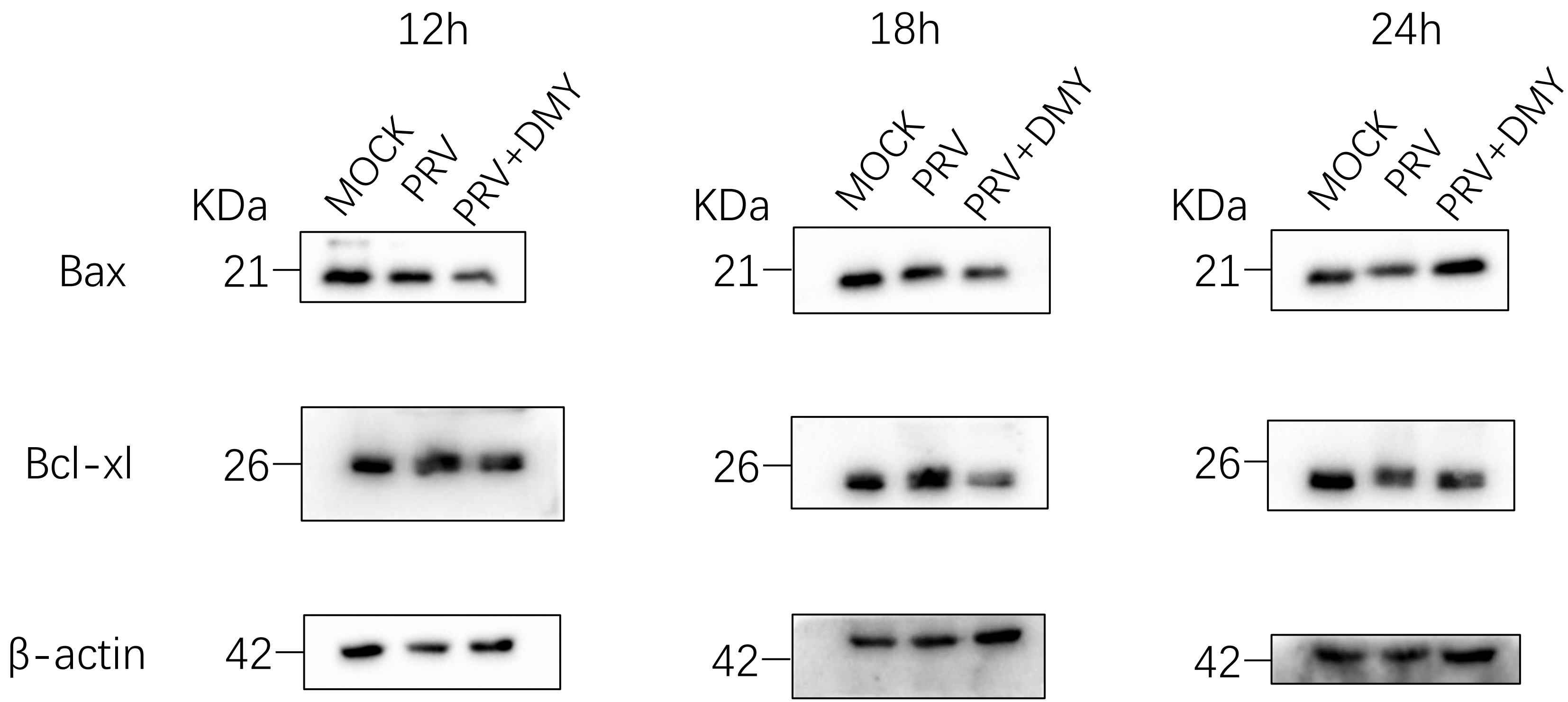

Figure S2.b

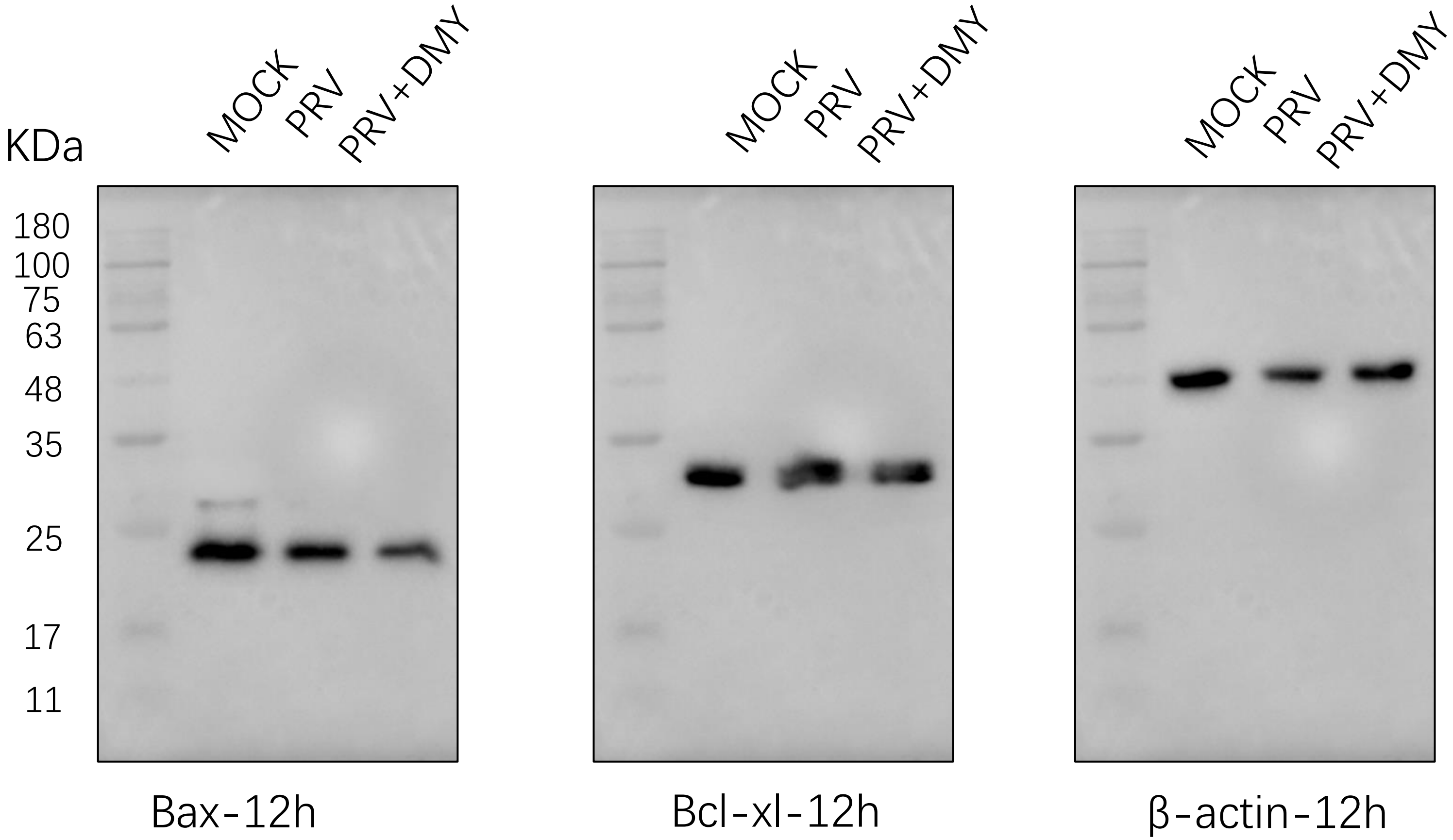
KDa
MOCK
PRV
PRV+DMY
180
100
75
63
48
35
25
17
11
Bax-12h
Bcl-xl-12h
β-actin-12h

Figure S2.c

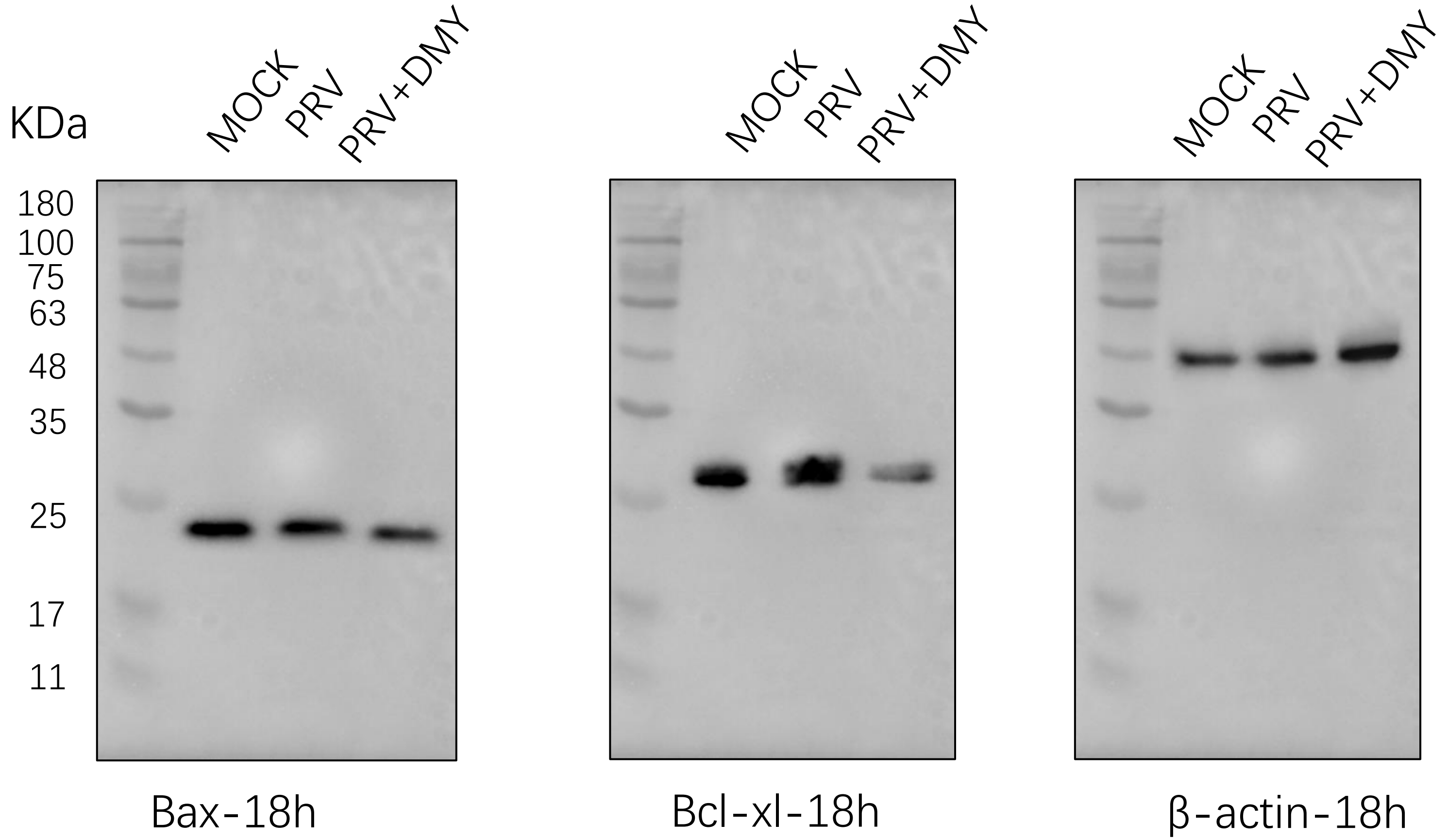
KDa
MOCK
PRV
PRV+DMY
180
100
75
63
48
35
25
17
11
Bax-18h
Bcl-xl-18h
β-actin-18h

Figure S2.d

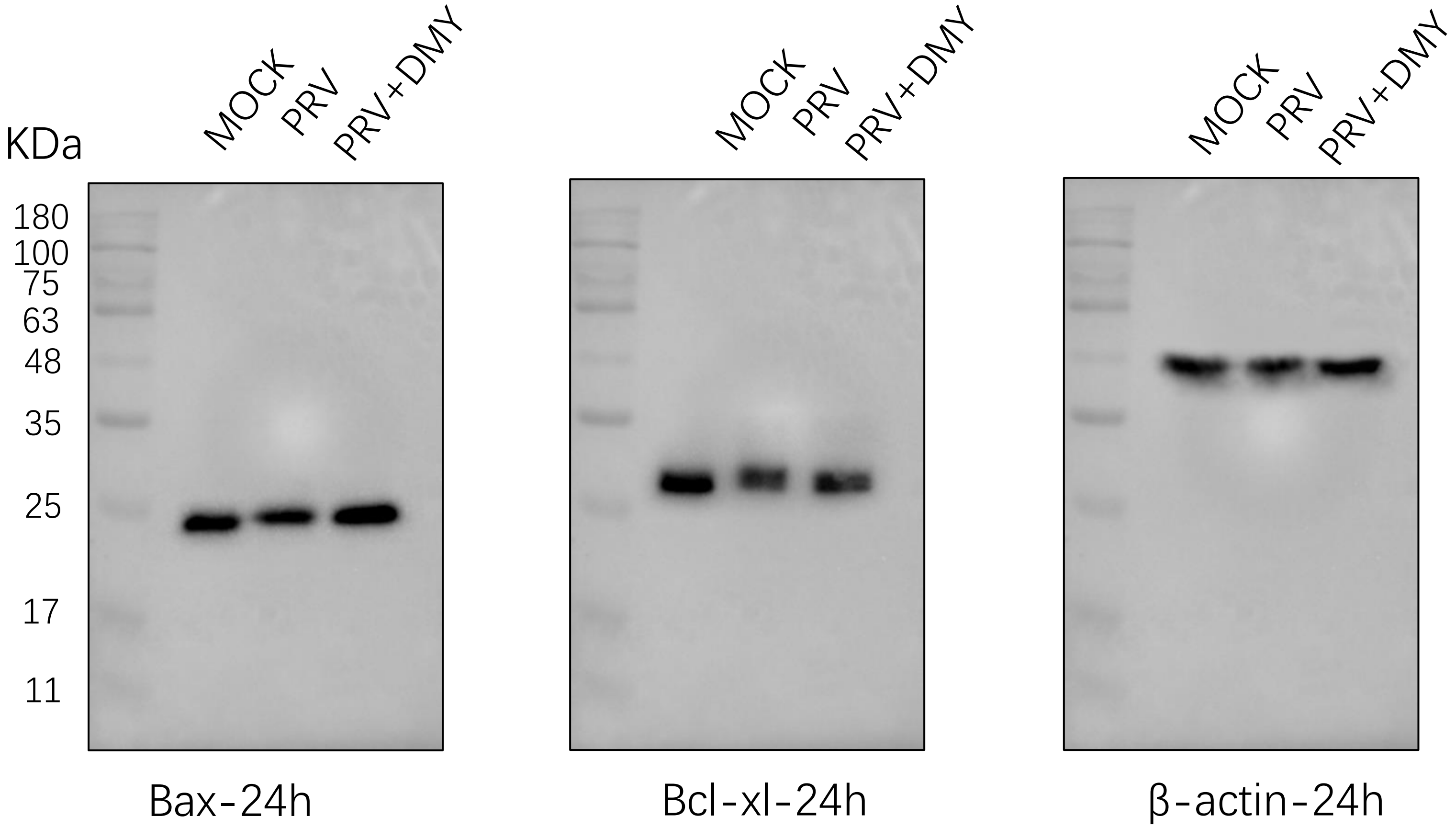
KDa
MOCK
PRV
PRV+DMY
180
100
75
63
48
35
25
17
11
MOCK
PRV
PRV+DMY
MOCK
PRV
PRV+DMY
Bax-24h
Bcl-xl-24h
β-actin-24h

Table S2: Intensity ratio of the Western blot bands in Figure 4

12h	Mock	PRV	PRV+DMY
Bax	1	0.84±0.02	0.54±0.01
Bcl-xl	1	1.47±0.01	0.62±0.02
β-actin	1	0.93±0.04	1.06±0.13

18h	Mock	PRV	PRV+DMY
Bax	1	0.79±0.01	0.53±0.01
Bcl-xl	1	1.30±0.02	0.67±0.01
β-actin	1	0.98±0.01	1.09±0.04

24h	Mock	PRV	PRV+DMY
Bax	1	0.78±0.01	1.23±0.02
Bcl-xl	1	0.80±0.01	0.51±0.01
β-actin	1	0.99±0.01	1.07±0.02
